# Differential expression of hypoxia-inducible factors related to the invasiveness of epithelial ovarian cancer

**DOI:** 10.1038/s41598-021-02400-1

**Published:** 2021-11-25

**Authors:** Ho-Jun Shih, Hsin-Fang Chang, Chi-Ling Chen, Pao-Ling Torng

**Affiliations:** 1 Graduate Institute of Clinical Medicine, College of Medicine, National Taiwan University, Taipei, Taiwan; 2 Department of Obstetrics and Gynecology, National Taiwan University Hospital, Taipei, Taiwan; 3 Graduate Institute of Epidemiology and Preventive Medicine, College of Public Health, National Taiwan University, Taipei, Taiwan; 4 Department of Surgery, National Taiwan University Hospital, Taipei, Taiwan; 5 Department of Obstetrics and Gynecology, Hsin-Chu Branch, National Taiwan University Hospital, Hsin-Chu, Taiwan

**Keywords:** Cancer, Ovarian cancer

## Abstract

Ovarian cancer is the most lethal gynecological cancer, and it is frequently diagnosed at advanced stages, with recurrences after treatments. Treatment failure and resistance are due to hypoxia-inducible factors (HIFs) activated by cancer cells adapt to hypoxia. IGFBP3, which was previously identified as a growth/invasion/metastasis suppressor of ovarian cancer, plays a key role in inhibiting tumor angiogenesis. Although IGFBP3 can effectively downregulate tumor proliferation and vasculogenesis, its effects are only transient. Tumors enter a hypoxic state when they grow large and without blood vessels; then, the tumor cells activate HIFs to regulate cell metabolism, proliferation, and induce vasculogenesis to adapt to hypoxic stress. After IGFBP3 was transiently expressed in highly invasive ovarian cancer cell line and heterotransplant on mice, the xenograft tumors demonstrated a transient growth arrest with de-vascularization, causing tumor cell hypoxia. Tumor re-proliferation was associated with early HIF-1α and later HIF-2α activations. Both HIF-1α and HIF-2α were related to IGFBP3 expressions. In the down-expression of IGFBP3 in xenograft tumors and transfectants, HIF-2α was the major activated protein. This study suggests that HIF-2α presentation is crucial in the switching of epithelial ovarian cancer from dormancy to proliferation states. In highly invasive cells, the cancer hallmarks associated with aggressiveness could be activated to escape from the growth restriction state.

## Introduction

Ovarian cancer is the most lethal gynecological cancer. The majority of malignant ovarian tumors are epithelial type ovarian cancer (EOC), which is diagnosed at advanced stages. Despite the aggressive surgical and chemotherapeutic treatments, the survival rate of patients with EOC is still low in most countries^1^. Rising death in EOC is due to the difficulty in early diagnosis and the treatment failure by unable to eradicate the cancer cells. Some cancer cells could survive from treatments due to their strong adaptation to the hypoxic environment developed from aggressive cancer growth or chemotherapy by activating hypoxia-inducible factors (HIFs)^2–4^. Hypoxia can modulate the cell response to cancer treatment and result in chemoresistance. Ovarian cancers are highly hypoxia-dependent, which affects the results of chemo- and immunotherapy. Therefore, alleviating hypoxia in ovarian cancer is essential for treatment efficacy. Targeting HIFs can help regulate angiogenesis, change the tumor microenvironment, increase the effect of chemotherapy, and help to overcome chemoresistance^5^.

The biological functions of hypoxia are associated with tumor progressions, such as angiogenesis^6^, epithelial-mesenchymal transition (EMT)^7^, invasiveness, metastasis^8^, and immune surveillance suppression^9^. It could also adversely affect the effects of chemotherapy with more unsatisfactory survival outcomes^10,11^. Many biological events associated with cancer cells occurred under a hypoxic tumor environment, such as the selection of clonal populations resistant to apoptosis^12^ and anaerobic metabolic switch ^13^. Intra-tumor hypoxia also contributes to genome instability by suppressing DNA-repair pathways^14^, DNA methylation^15^, and generating reactive oxygen species^16^. Many studies have focused on these fields to identify new hypoxia targets to assist the limitations of cancer treatments^17^.

HIFs play a central role in cellular adaptation to hypoxia. Mammals possess three isoforms of HIFα^18^. HIF-1α and HIF-2α overexpressions are driven by intra-tumoral hypoxia, growth factor signaling, and genetic mutations in oncogenes and tumor suppressor genes^19^. These are also associated with increased tumor vascularization and poor prognosis of various cancers such as breast, ovarian, and non-small cell lung^20^.

We have previously established an EOC cell line and several sublines with different invasion abilities. Insulin-like growth factor binding protein-3 (IGFBP3) was found as an invasion suppressor that negatively correlated to the invasiveness of EOC cell line. In the animal model, a low IGFBP3 expression was associated with a more significant tumor progression, higher cancer invasion, and distant metastasis, while a high IGFBP3 expression was associated with tumor necrosis and apoptosis^21^. IGFBP3 could also activate Thrombospondin-1 (THBS1), an anti-angiogenic protein, to inhibit tumor angiogenesis, resulting in a temporary tumor growth arrest^22^.

The lack of neovascularization during tumor growth causes tumor hypoxia. In this study, we explored the relationship between invasion and hypoxia in ovarian cancer cells. We also focused on biological events during tumor hypoxia and how cancer cells overcome the hypoxic microenvironment and proceed to tumor progression.

## Results

### Tumor regeneration from growth arrest under the expression of IGFBP3

**Figure 1:**
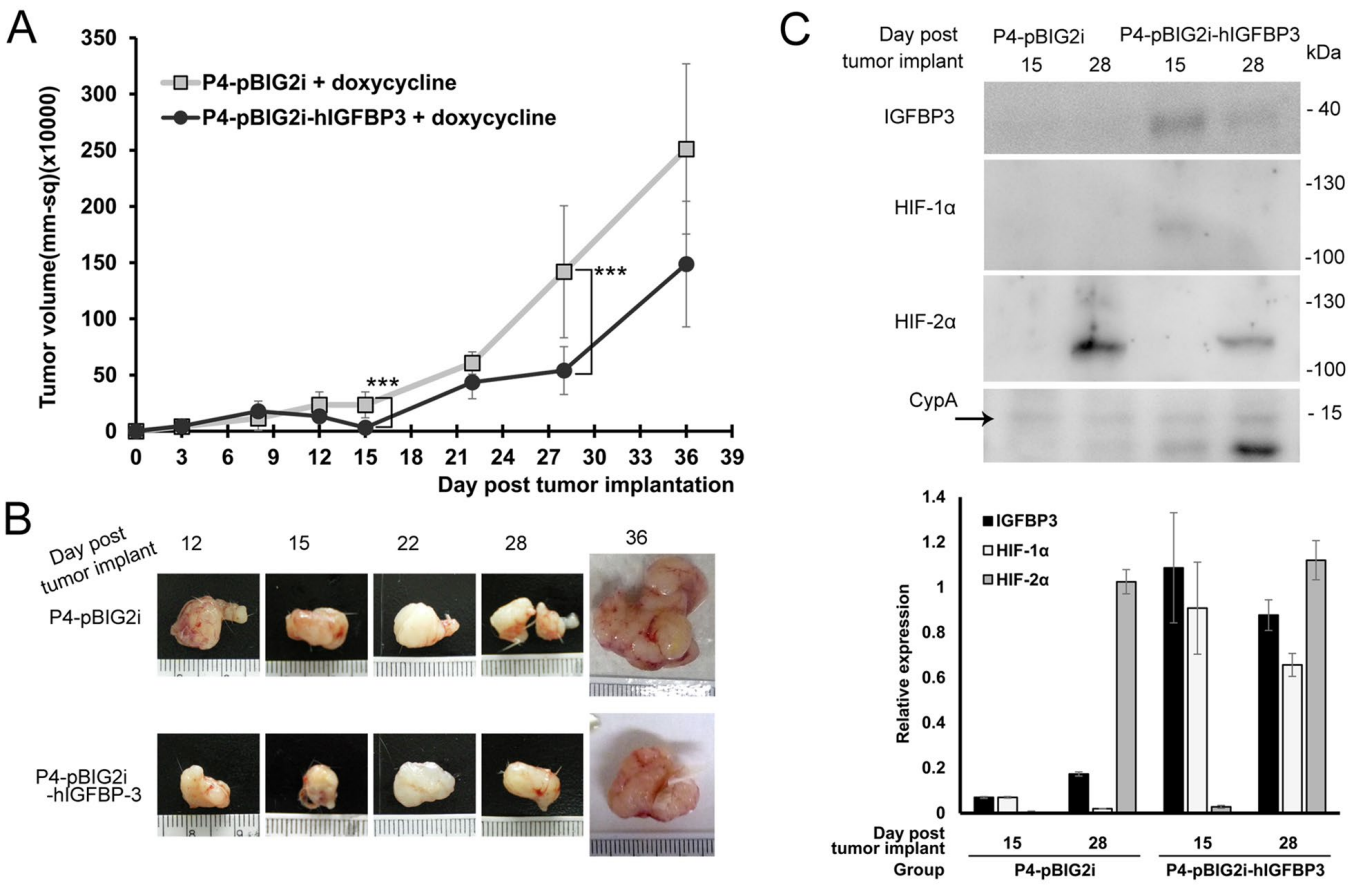
IGFBP3 expression inhibited tumor growth and created a hypoxic environment, which induced HIF-2α to overcome the oxygen stress. (**A**) Heterotransplantation of P4-pBIG2i and P4-pBIG2i-hIGFBP3 in SCID mice. Doxycycline was started to stimulate IGFBP3 at 8 d. Xenograft tumor sizes were measured, and the mice were sacrificed at 12, 15, 22, 28, and 36 d after the implantation. (Five mice per group in each time point. The error bars represent the SDs; ***P < 0.0005.) (**B**) Photographs of xenograft tumors. (**C**) Western blot analysis and signal quantitative detections of IGFBP3, HIF-1α, and HIF-2α expressions in xenograft tumors at 15 and 28 d. Cyclophilin A (CypA) as the loading control. The signals of Western blot were quantified and analyzed by Image Studio Lite version 5.2. Excel 2016 was used to generate charts. Photoshop CS2 version 9 was used to assemble the figure.

The ovarian cell line, OVTW59-P0, was established from an EOC, endometrioid adenocarcinoma, and its subline P4 was selected sequentially from Matrigel-coated transwell membranes. We found P0 expresses IGFBP3, is less migrative, invasive and metastatic than P4^21^. In addition, P0 inhibits angiogenesis through intracellular regulation of THBS1 expression, an angiogenesis inhibitor^22^. In our previous study, xenografts with IGFBP3-expressing cell lines, P0, and P4-I (P4-pKG3226-hIGFBP3, a stable P4 transfectants expressing IGFBP3) showed growth arrest associated with the inhibition of vasculogenesis and tumor necrosis. This was not observed in xenografts with very low IGFBP3-expressing cell lines, P4, and P4-V (P4-pKG3226, a stable P4 transfectants without IGFBP3)^21,22^. A doxycycline-inducible-expressing plasmid, pBIG2i-hIGFBP3, was established to study the chronological reactions after IGFBP3 expression. Breeding P4-pBIG2i-hIGFBP3 or control P4-pBIG2i transfectants on SCID mice, tumor growth was found to be attenuated after IGFBP3 expression. However, the tumors started to grow again after 15 d of tumor cell implantation (IGFBP3 was continuously stimulated for expression for 7 d). Nevertheless, the growth of P4-pBIG2i-hIGFBP3 was retarded compared to that of P4-pBIG2i (Fig. 1A,B).

### Early HIF-1α and late HIF-2α expressions related to IGFBP3

To analyze how tumors overcome the growth suppressive conditions and restart to grow, we examined the expressions of HIF-1α, and HIF-2α, in these xenografts at 15 and 28 d, when the tumors had the most significant differences in volume (Fig. 1C). In P4-pBIG2i, HIF-1α was not expressed on both days, while HIF-2α was highly expressed at 28 d. In P4-pBIG2i-hIGFBP3, HIF-1α was strongly expressed at 15 d, but it was slightly reduced at 28 d, while HIF-2α was weakly expressed at 28 d. These results suggest that HIF-1α was expressed during tumor arrest mediated by IGFBP3. The results show that IGFBP3 was associated with high HIF-1α and low HIF-2α expressions in prolonged hypoxia. The expression of HIF-2α persisted in growing tumor cells without IGFBP3 (P4-pBIG2i group).

**Figure 2:**
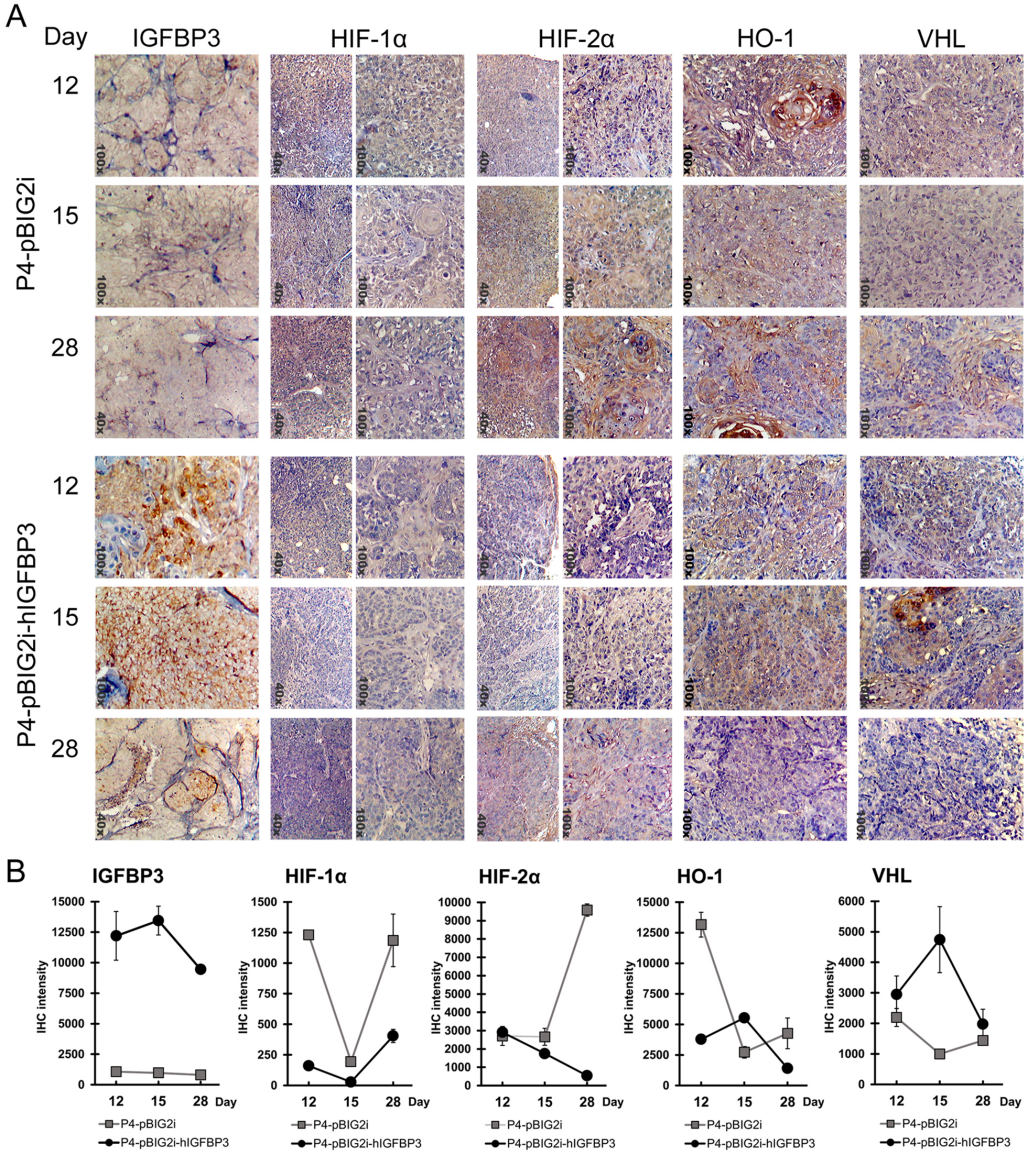
Immunohistochemistry staining of xenografts on 12, 15, and 28 d after the tumor implantation. (**A**) Heterotransplantation tumors P4-pBIG2i and P4-pBIG2i-hIGFBP3 at 12, 15, and 28 d post-tumor implantation on IGFBP3 (100 ×), HIF-1α (40 × and 100 ×), HIF-2α (40 × and 100 ×), HO-1 (100 ×), and VHL (100 ×). P4-pBIG2i xenograft showed no IGFBP3 expressions, while P4-pBIG2i-hIGFBP3 showed a strong IGFBP3 expression after adding doxycycline (started on d 8). HIF-2α increased over time in P4-pBIG2i xenograft, but it only accumulated after 28 d in P4-pBIG2i-hIGFBP3 xenograft. Xenograft tumors without IGFBP3 expressed HIF-2α majorly, especially under a prolonged tumor growth in hypoxic environments. (**B**) Quantitative analysis of immunohistochemical staining. Images of IHC were taken by Motic Image Plus 3.0 software. The signals of IHC were quantified and analyzed by ImageJ 1.53 k software. Excel 2016 was used to generate charts. Photoshop CS2 version 9 was used to assemble the figure.

Using Immunohistochemistry (IHC) staining, these HIF changes became more apparent. The xenograft’s protein expressions at 12, 15, and 28 d after implantation were analyzed (Fig. 2A,B). In the IGFBP3-present group (P4-pBIG2i-hIGFBP3 xenograft), IGFBP3 was strongly expressed at 12 and 15 d and less expressed at 28 d. HIF-1α and HIF-2α showed similar and feeble expressions. However, in the group without IGFBP3 (P4-pBIG2i xenograft), HIF-1α was less expressed, while HIF-2α accumulated at the beginning, increased gradually, and became strongly expressed at 28 d.

The expressions of Heme Oxygenase-1 (HO-1) and Von Hippel-Lindau (VHL) were also analyzed. HO-1 is stimulated by hypoxia and is induced by HIFs^23^, while VHL is a tumor suppressor protein that regulates HIFs’ activities and is increased under hypoxia^24^. Both HO-1 and VHL were expressed in P4-pBIG2i and P4-pBIG2i-hIGFBP3 xenografts. In P4-pBIG2i cells, the expressions of HO-1 and VHL increased in parallel with HIF-2α. In P4-pBIG2i-hIGFBP3 cells, HO-1 and VHL were highly expressed at 15 d when the tumor was the smallest and then subsided at 28 d when the tumor started to grow again.

Overall, the Western blot and IHC results showed that HIFs accumulated when the tumor entered a hypoxic phase. In the transplantation with/without low IGFBP3 expression, a continuous expression of HIF-2α allows the tumor to escape from the growth arrest caused by hypoxia.

### Hypoxia induces HIF-1α but not HIF-2α in cells with less invasive capabilities

To confirm the HIF patterns associated with IGFBP3 and related to invasive capabilities, the HIF expressions were studied in these cell lines using a hypoxia chamber. P0 and P4 were cultured on glass slides, and these cells were incubated under normoxia and hypoxia (1.0% O_2_ and 5% CO_2_, 37 °C, 17 h) conditions. Then, the expressions of IGFBP3, HIF-1α, and HIF-2α were analyzed. A549, which is known to express HIF-2α majorly, was cultured as a hypoxic condition and HIF-2α expression control.

**Figure 3:**
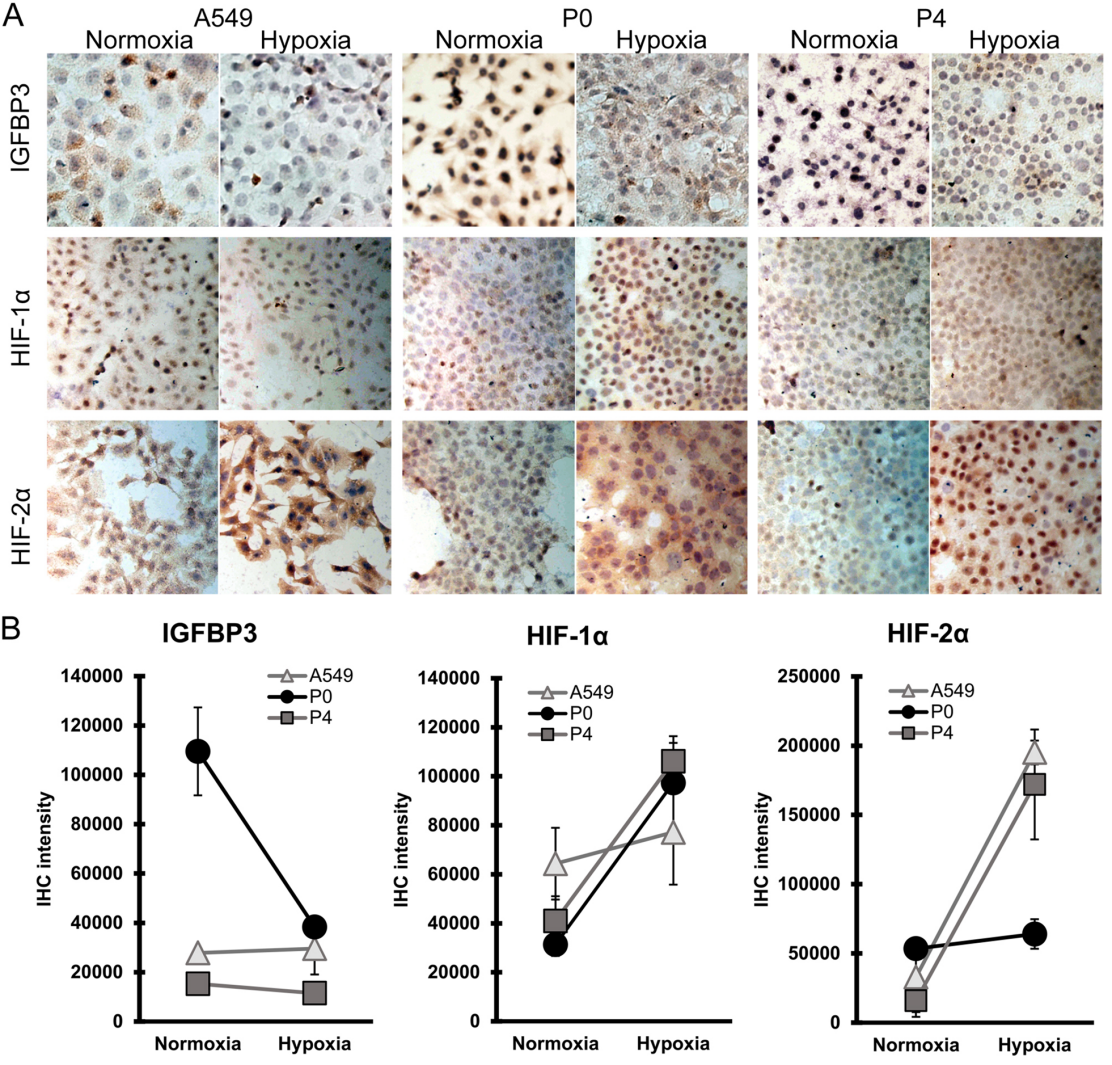
Immunocytochemistry staining of A549, P0, and P4 cell cultures under normoxic and hypoxic conditions for 17 h. (**A**) Under normoxic conditions, P0 expressed more IGFBP3 than P4. P0 showed an increase in HIF-1α, while P4 showed a higher increase in HIF-2α under hypoxia than that under normoxia, indicating that tumor cells without IGFBP3 induced more HIF-2α under acute hypoxic conditions. The A549 cell culture that expressed HIF-2α under hypoxic conditions was used as the study control. (**B**) Quantitative analysis immunohistochemical staining. Images of ICC were taken by Motic Image Plus 3.0 software. The signals of IHC were quantified and analyzed by ImageJ software. Excel 2016 was used to generate charts. Photoshop CS2 version 9 was used to assemble the figure.

**Figure 4:**
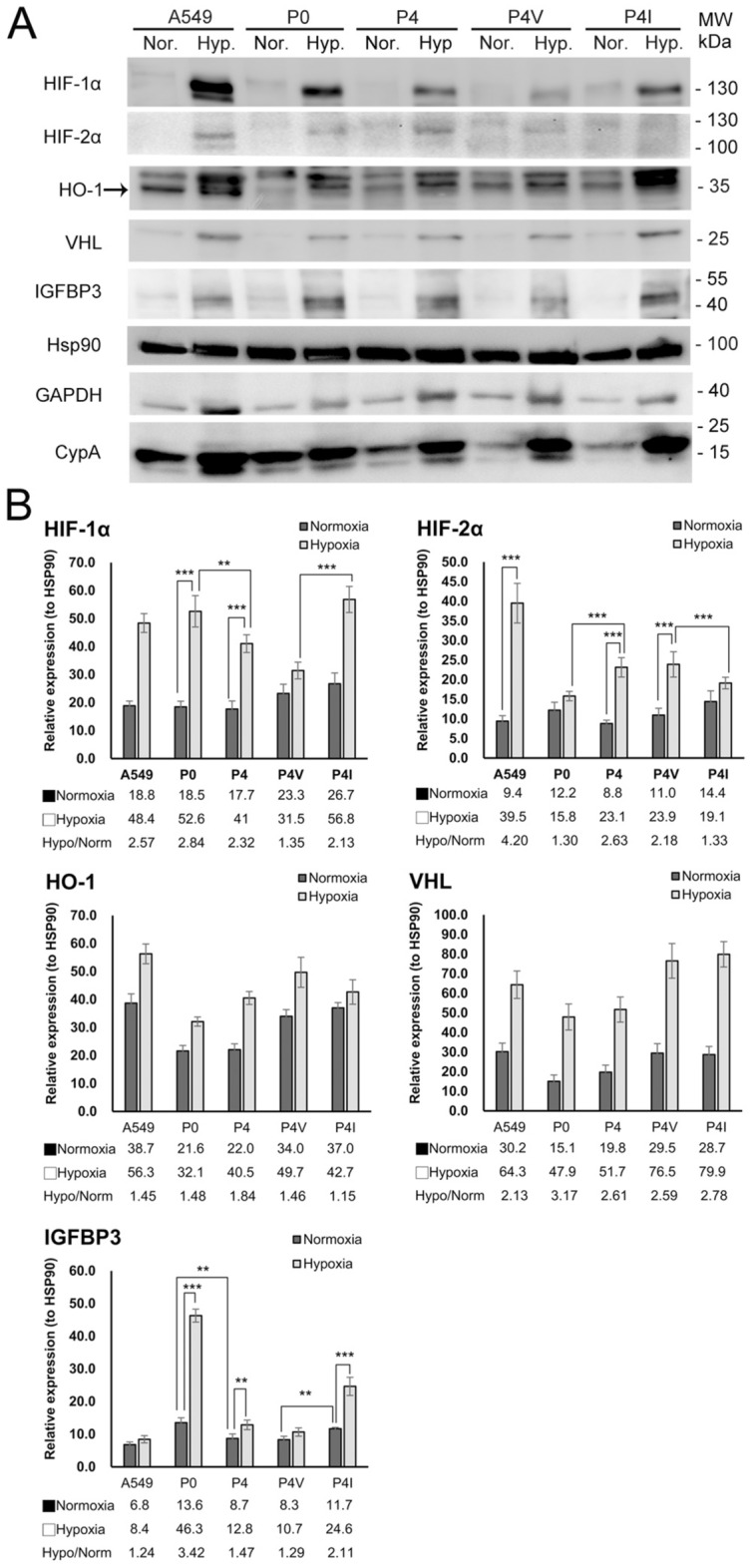
Protein expression analysis of cell cultures under normoxic and hypoxic conditions. (**A**) Western blot analysis of IGFBP3, HIF-1α and HIF-2α, HO-1, VHL, and GAPDH expressions in P0, P4, P4 transfectants, and A549 under normoxic and hypoxic conditions. HSP90 was added as the control. (**B**) Western blot results were transformed into bar figures using Image Studio Lite version 5.2. Signal strengths were quantified and normalized with HSP90. P0 had a higher IGFBP3 expression than P4. In P0 and P4-I, IGFBP3 expression was increased under hypoxia. Accumulation of HIF-1α or HIF-2α correlated with IGFBP3 expressions: a higher IGFBP3 was with a stronger HIF-1α. Both HO-1 and VHL expressions were increased under hypoxia. Each sample was assayed in triplicates, and the experiment was repeated three times independently. (The error bars represent the SDs; **P < 0.001, ***P < 0.0005). Excel 2016 was used to generate charts. Photoshop CS2 version 9 was used to assemble the figure.

Immunocytochemistry (ICC) staining showed that A549 highly expressed HIF-2α, P0 had an increase in HIF-1α, while P4 had a markedly increase in HIF-2α under hypoxia (Fig. 3A,B). Based on Western blot (Fig. 4A, signals quantified in Fig. 4B), both P0 and P4-I (i.e., P4-pKG3226-hIGFBP3) showed an increase in IGFBP3 and expressed HIF-1α more than HIF-2α under hypoxia. P4 and P4-V (i.e., P4-pKG3226) showed a slight increase in IGFBP3, and HIF-2α was markedly activated under hypoxia compared with HIF-1α. HSP90 was used as a control because GAPDH, cyclophilin A, and vinculin (data not shown) were increased under hypoxia in cancer cells^25,26^. Both VHL and HO-1 were expressed under normoxia and increased under hypoxia. These results suggest that the function and the synthesis pathway of HIFs were operating effectively.

### Regulation of HIF synthesis in P0 and P4 under hypoxia

**Figure 5:**
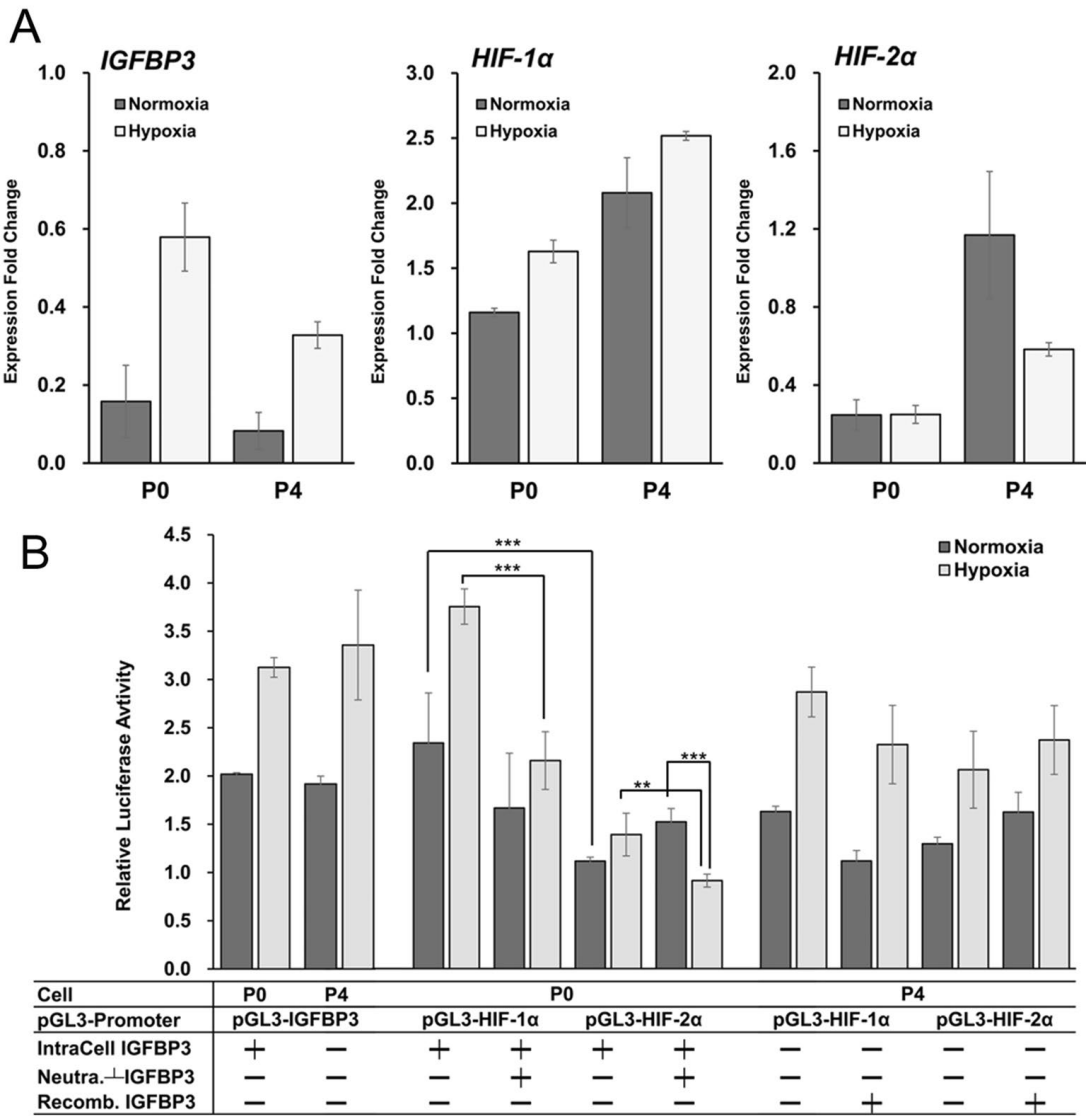
IGFBP3 differentially regulated HIF-1α and HIF-2α synthesis in low and high invasion ability cells. Gene expression and regulation of IGFBP3, HIF-1α, and HIF-2α in cells cultured under normoxic and hypoxic conditions. (**A**) qPCR analyzed of IGFBP3, HIF-1α, and HIF-2α mRNA expressions in P0 and P4 cultured under normoxic and hypoxic conditions. GAPDH as the normalized control. P4 expressed higher *HIF-1α* and *HIF-2α* under normoxia, and P0 expressed lower *HIF-2α* compare with P4. (**B**) Luciferase promoter assays on the regulations of IGFBP3, HIF-1α, and HIF-2α promoters in P0 and P4 under normoxic and hypoxic conditions. The table below indicates the presence of endogenous or exogenous IGFBP3. In high-IGFBP3 expression P0, HIF-1α was more activated than HIF-2α at hypoxia. Both HIF-1α and HIF-2α were reduced after depletion of extracellular IGFBP3 by neutralizing antibodies (lanes 4 and 6). In low-IGFBP3 expression P4, both HIF-1α and HIF-2α activities increased under hypoxia. There were no changes in HIF-1α and HIF-2α expressions after adding recombinant hIGFBP3 (lanes 8 and 10). Each sample was assayed and analyzed repeated three times independently. (The error bars represent the SDs; **P < 0.001, ***P < 0.0005.). Excel 2016 was used to generate charts. Photoshop CS2 version 9 was used to assemble the figure.

We analyzed the IGFBP3, HIF-1α, and HIF-2α mRNA expressions in P0 and P4 under normoxia and hypoxia using GAPDH as a control^27^. As shown in Fig. 5A, P0 had a higher *IGFBP3* expression than P4 in both conditions. *IGFBP3* and *HIF-1α* expressions were increased under hypoxia in both cells. P4 expressed higher *HIF-1α* and *HIF-2α* under normoxia, while P0 had a significantly lower *HIF-2α* expression compare to P4 in both conditions.

Luciferase promoter assay was performed to study the effects of IGFBP3 and hypoxia on HIF-1α and HIF-2α expressions in cells with low (P0) or high (P4) invasion-metastasis abilities (Fig. 5B). IGFBP3 promoters were activated in P0 and P4. In P0, HIF-1α was more activated than HIF-2α. After adding IGFBP3-neutralizing antibodies to deplete extracellular IGFBP3, HIF-1α activations were more reduced than HIF-2α activations under hypoxia. These suggest that IGFBP3 could interact more efficiently with HIF-1α but not with HIF-2α. In P4, both HIF-1α and HIF-2α activities were increased under hypoxia. There was no specific change in HIF-1α and HIF-2α activities after adding the recombinant hIGFBP3. This suggests that cancer cells with more invasive abilities and low IGFBP3 tend to activate HIF-2α.

These suggest that hypoxia itself could increase the synthesis of IGFBP3, which could also regulate HIF synthesis and activate cells with low invasion-metastasis capabilities.

### Angiogenesis-related protein expressions under oxygen stress

**Figure 6:**
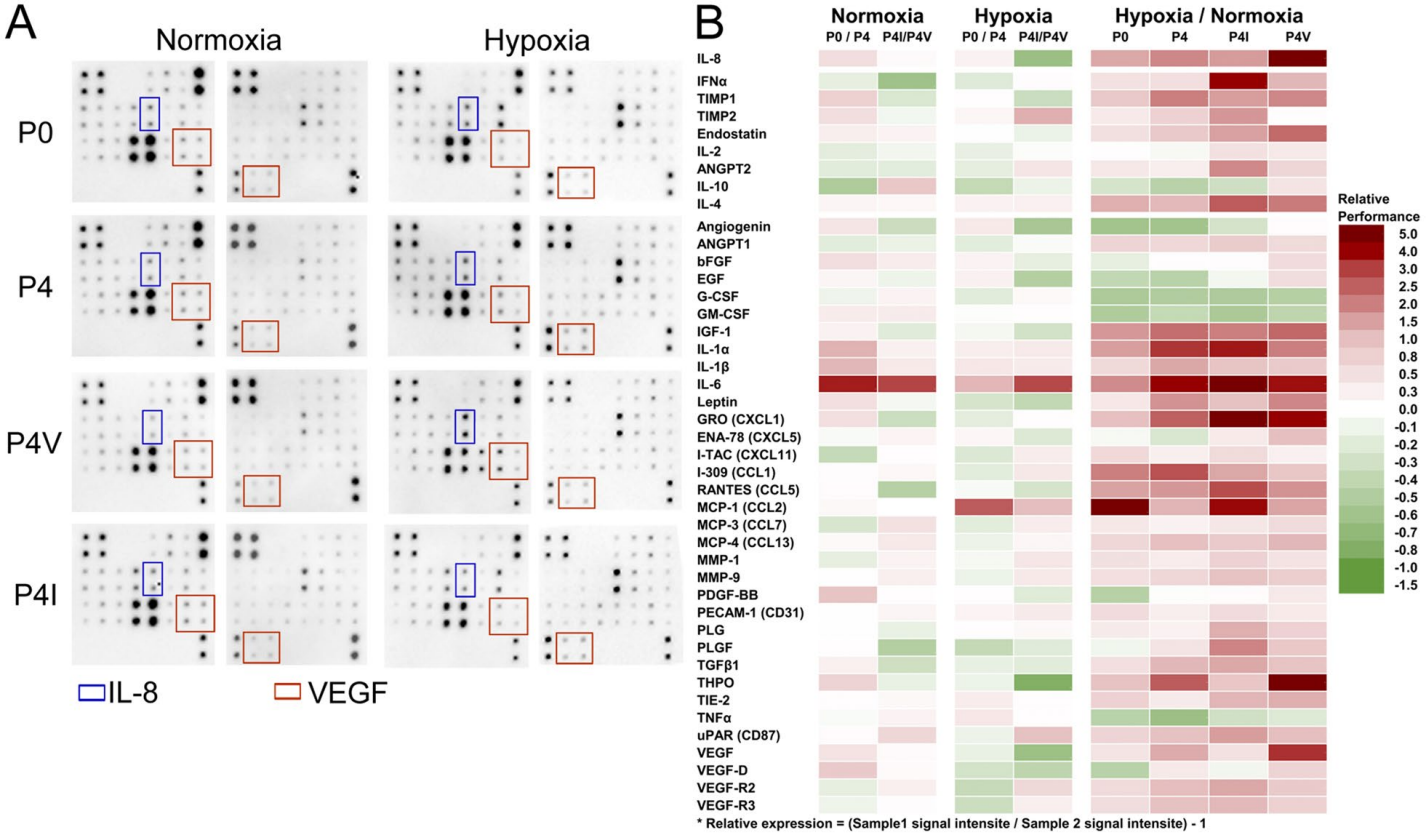
Low IGFBP3 expression is associated with a high expression of angiogenic proteins related to HIF-2α under hypoxia. (**A**) Dot data of angiogenesis antibody array in P0, P4, P4-V, and P4-I lysates under normoxia and hypoxia. IL-8 showed a similar expression pattern as VEGF. Both IL-8 and VEGF were more expressed under hypoxia than in normoxia. (**B**) Expression signals were presented in categories by importance related to hypoxia and functions related to vasculogenesis. (IL-8 was negatively correlated with HIF-1α and positively correlated with HIF-2α. The proteins listed: IFNα to IL-4 are proteins with anti-angiogenic abilities; angiogenin to VEGF-R3 are known as angiogenic proteins.) The dot signals were analyzed with Image Studio Lite version 5.2, and the relative expression levels were compared between the groups. Red represents a higher signal performance, while green represents a lower signal performance than the comparison cell. Excel 2016 was used to generate chart. Photoshop CS2 version 9 was used to assemble the figure.

IGFBP3 could inhibit angiogenesis through THBS1 expression^22^. Abcam’s angiogenesis antibody array was used to identify more targets involved in the production of different HIFs under normoxic and hypoxic conditions. Figure 6 shows that P4 expressed a more substantial amount of angiogenic proteins, VEGFs and VEGFRs for example (Fig. 6A, red frame), than P0. P4 also expressed more proteins with substantial angiogenic potential than P0 under hypoxic conditions (Fig. 6A quantitative result in Fig. 6B, block: hypoxia, row: P0/P4), suggesting that P4 has a stronger angiogenic protein reaction to compensate for hypoxic conditions compared to P0. More importantly, P4 and P4-V had a more robust signal on IL-8 compare to P0 and P4-I (Fig. 6A, blue frame). A study reported that the expression of IL-8 was decreased in the presence of HIF-1α but increased in the presence of HIF-2α^28^. Our experiments show the predominant expression of HIF-1α in P0 and P4-I and that of HIF-2α in P4 and P4-V. Therefore, it is reasonable to infer that P4 induced HIF-2α under hypoxia stress.

This study showed that re-expressing IGFBP3 could transiently suppress tumor growth. The switch from growth inhibition to proliferation occurred by activating HIFs, such as HIF-1α or HIF-2α.

## Discussion

IGFBP3 was previously identified as an invasion/metastasis and growth suppressor. IGFBP3 could inhibit angiogenesis by upregulating THBS1, leading to tumor growth arrest. Tumor cells eventually overcame growth arrest status and switched to tumor proliferation after long days. Here, HIF-2α was identified as a critical protein that displays the switch from tumor growth arrest to tumor proliferation.

Different isoforms of HIFs were activated in our cell lines with different invasive capabilities. HIF-1α was activated in P0 and IGFBP3-re-expressing P4. HIF-1α and HIF-2α were both activated in P4 and P4 vector-only transfectants. These results imply that the activation of HIF-1α and HIF-2α could be associated with different hallmarks of cancer aggressiveness.

It was reported that HIF-1α and HIF-2α respond to different hypoxia condition, HIF-1α mediates the acute response (< 24 h), while HIF-2α regulates the cellular response under prolonged hypoxia^29^. HIF-1α is expressed in smooth muscle cells surrounding the blood vessels^30^ and is associated with angiogenesis, EMT, cancer metabolism, invasion, and metastasis^31–33^. In contrast, HIF-2α is expressed in endothelial cells of blood vessels^30^, kidney and lung epithelial cells, bone marrow macrophages, and neural crest derivatives during development^34^. HIF-2α is also known to induce angiogenesis, vasomotor, and upregulate many transcription factors in cancer^35^.

Ovarian tissues commonly express HIF-1α, which could play an important role in the cyclic angiogenesis necessary in developing ovarian follicles and corpus luteum^36^. Some studies showed that the overexpression of HIF-1α was associated with poor survival in patients with EOC^37^. A high expression of HIF-2α was reported to correlate with poor prognosis and high clinical stage^38^. Both HIF-1α and HIF-2α seemed to be equally crucial in cancer invasion and aggressiveness^39^.

**Figure 7:**
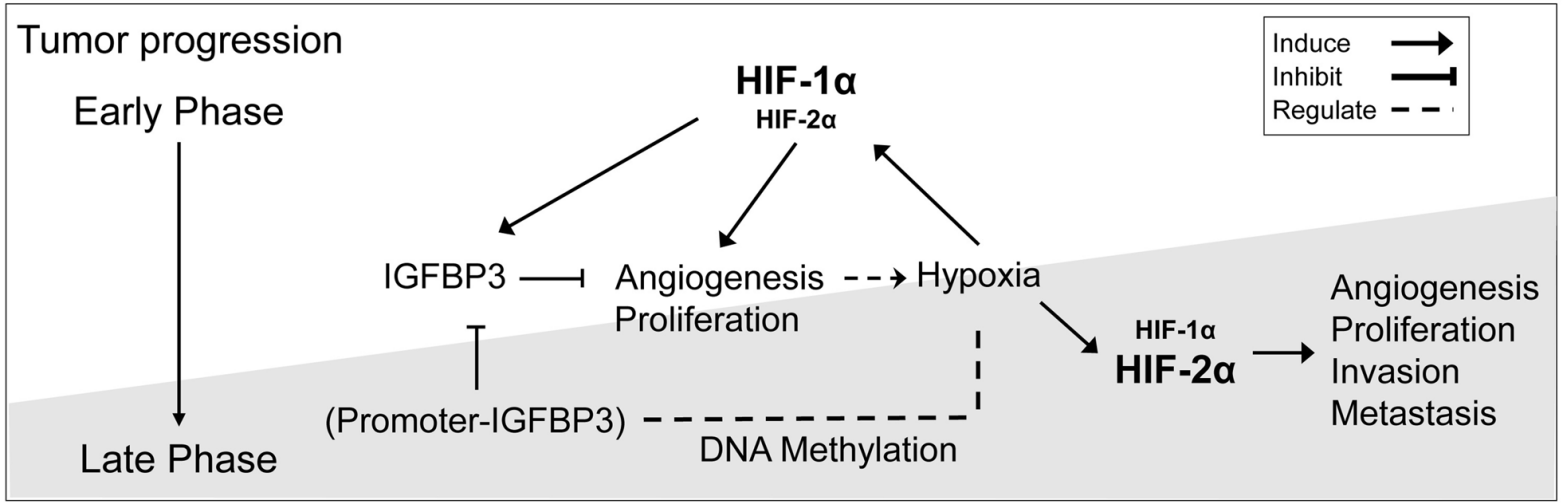
Clinical roles of HIF-1α and HIF-2α related to IGFBP3 expression in EOC. In an early stage of EOC, IGFBP3 expresses and inhibits proliferation and angiogenesis. The lack of blood vessels causes tumors to enter a hypoxic state, which stimulates cells to activate HIFs. HIF-1α is the major HIFs in this tumor stage. HIF-1α stimulates angiogenesis, proliferation, and induces IGFBP3 synthesis. The continuous expression of IGFBP3 prolongs the hypoxic state of the tumors by inhibiting tumor vasculogenesis. Prolonged hypoxia causes the promoter to be methylated and silence the expression of IGFBP3. As IGFBP3 decreases, the cells begin to proliferate and switch to activate HIF-2α majorly under hypoxic conditions. HIF-2α plays a significant role in cancer aggressiveness by regulating angiogenesis, invasion, and metastasis. Henceforth, the cancer cells mainly express HIF-2α under hypoxic stress. The decrease in IGFBP3 and the activation of HIF-2α instead of HIF-1α accelerate the progression in EOC. PowerPoint 2016 and Photoshop CS2 version 9 were used to generate figure.

The unclear clinical significance of HIFs in EOC could be due to the heterogeneity of the composition of cancer tumors and late hypoxic events. This study is unique because it involved cancer cells with a known degree of invasiveness, which is correlated with IGFBP3, and the studies of hypoxic events were in a chronological sequence. Therefore, we could clarify the clinical roles of IGFBP3, HIF-1α and HIF-2α in EOC progression: HIF-1α being important in early hypoxia response and HIF-2α playing in prolonged hypoxia that could be associated with the overcome of tumor growth restriction (Fig. 7). In addition, IGFBP3 is inactivated through promoter methylation and results in EOC progression^40^. The hypoxic environment causes the promoter to be methylated^15^ and reduces the expression of IGFBP3. The decrease in IGFBP3 and the activation of HIF-2α instead of HIF-1α accelerate the progression in EOC.

The mechanism of how low IGFBP3 expression increase HIF-2α is unclear. It was reported that insulin, the insulin-like growth factor (IGF), IGFPB and HIF could regulate each other in a reciprocal way. Insulin, IGF-1, and IGF-2 induce expression of HIF-1α, and HIF-1α regulated the expression of IGF-2, IGFBP2, and IGFBP3 under hypoxia^41^. IGFBP3 is reported as a direct HIF-1α and HIF-2α target gene and is highly translated under hypoxic conditions^42,43^. HIFs transactivates IGFBP3 through the hypoxia response element (HRE), the sequence recognized by HIFs, in the promoter of *IGFBP3*^41^*.*

This study shows the increase in IGFBP3 promoter activity, mRNA, and protein expression under hypoxia, suggesting that the hypoxia response element (HRE) of IGFBP3 functions normally and that the regulatory function of HIFs is normal. HO-1 and VHL have an increased expression under hypoxia^23,24^, as shown in this study. These clarified that the results showing the shift from HIF-1α to HIF-2α is not through the loss of function in the HIF pathway.

The chronological sequence of HIF changes could be more closely observed in IHC and ICC studies in Figs. 2 and 3. In the presence of IGFBP3, there was no HIF-2α accumulation under chronic hypoxia (Fig. 2). Without IGFBP3, HIF-1α responds weakly to acute hypoxia (Fig. 3) and then shifted to HIF-2α in prolonged hypoxia (Fig. 2). These suggest that this HIF shift could be related to cells with different invasion-metastasis abilities.

Targeted therapy, in additive to current chemotherapeutic regimens, much improved survival outcome in advanced stage ovarian cancer. One of the target drugs currently used is bevacizumab, which inhibits angiogenesis^44,45^. Many studies have focused on the pathways related to hypoxia. Lacking of blood vessels causing tumors to enter a hypoxic state, and activates HIFs to overcome the hypoxic stress, resulting in treatment resistance. Therefore, drugs targeting on HIF-2α, such as PT2358^46^ and Belzutifan^47^, to be potential new drugs for ovarian cancer treatment.

Our research shows that the activation of HIF-1α or HIF-2α by cancer cells is related to the invasion ability of ovarian cancer cells. Highly aggressive ovarian cancer cells show lower IGFBP3 and tend to express HIF-2α under hypoxic conditions. The expression patterns of IGFBP3 and HIFs can be used in the future as diagnostic markers on the invasiveness of ovarian cancer, and on the design for the strategy of target therapy.

This study provides a cell model as a basis for future studies on the chronological sequence of HIF changes and effects on tumor cell functions, and in clinically, provides new candidate for target therapy. More studies are needed to determine the mechanisms of the shift between HIF-1α and HIF-2α in tumorigenesis.

## Conclusions

In conclusion, this study showed the importance of HIF-1α and HIF-2α in tumor growth arrest and proliferation. HIF-1α could react with IGFBP3 associated with tumor arrest signals, while HIF-2α assists tumor growth and aggressiveness. Highly aggressive cancer cells have more cancer hallmarks to escape from the growth restriction states. This study also provides more detailed events during tumor hypoxia and supports the development of a better diagnostic and treatment for EOC.

## Material and methods

### Cell lines and plasmids

The EOC cell line OVTW59 P0 and P4 sublines (human, established in our lab)^21^ were maintained in a DMEM solution with 5% FBS. P0 was the original OVTW59 cell line, while P4 was a subline selected sequentially from P0, was more invasive than P0. The A549 cell line (human, kindly, provided by Dr. Lin from National Taiwan University Hospital, NTUH), which expresses HIF-2α under hypoxic conditions, was used as the study control.

In the inducible IGFBP3-expressing cell line, which was used only in the in vivo study, the full-length human IGFBP3 cDNA was constructed in a doxycycline-induced expression plasmid pBIG2i (provided by Dr. Lin from NTUH) and was labeled as -pBIG2i-hIGFBP3. Transfectants with plasmids without the IGFBP3 cDNA were labeled as -pBIG2i. IGFBP3 expression was induced by 2 mg/mL of doxycycline in drinking water during heterotransplantation. In the constant IGFBP3-expressing cell line, the full-length human IGFBP3 cDNA was constructed in the expression vector pKG3226 that contains the human β-actin promoter and was labeled as -pKG3226-hIGFBP3 (-I)^48^ . Transfectants with plasmid without the IGFBP3 cDNA was labeled as -pKG3226 (-V). The transfection of the plasmids followed the protocol of Arrest-In Transfection Reagent (Open Biosystems, Inc. Huntsville, AL, USA).

### Tumorigenicity of P4-pBIG2i-hIGFBP3 and P4-pBIG2i cells in SCID mice

Severe combined immunodeficiency (SCID) mice, aged 6–8 weeks, were transplanted subcutaneously with 2 × 10^7^ of P4-pBIG2i-hIGFBP3 or P4-pBIG2i-transfected cells. The sizes of tumor growth were measured using vernier calipers. The formula to calculate the tumor volume: length × width × width × 0.52, approximates a solid elliptical mass volume.

In addition, 2 mg/mL of doxycycline and 2% sucrose were added in the drinking water of animals, starting on d 8 when the diameter of the tumor exceeded 0.5 cm. The mice were autopsied on 12, 15, 28, and 36 d after the tumor implantation, and the xenograft tumors were examined.

Female SCID mice were weighed and randomly divided into two groups, transplanted subcutaneously either with P4-pBIG2i-hIGFBP3 or with P4-pBIG2i-transfected cells. Five mice in each group were sacrificed on 12, 15, 28, and 36 days after tumor implantation (or at humane endpoints) by cervical dislocation after anesthesia by Isoflurane inhalation. The primary humane endpoints selected in the study were weight loss, reduction of activities, the presence of open wounds at heterotransplantation site, infection, or behavior change. All experimental procedures were approved by the Institutional Animal Care and Use Committee (IACUC) at National Taiwan University College of Medicine and College of Public Health (IACUC Approval No: 20130524). All methods were carried out in accordance with IACUC guidelines and regulations. This study was carried out in compliance with the ARRIVE guidelines.

### Hypoxic stimulation

The tumor cells were cultured in dishes, well plates, and glass slides under normoxic conditions until 70% confidence; then, these were moved to a hypoxia chamber containing 1.0% of O_2_ and 5% CO_2_ for 17 h.

### Immunohistochemistry (IHC) and immunocytochemistry (ICC)

In IHC, paraffin blocks of the xenograft tumors were cut into 4-μm sections. The slides were incubated with primary antibodies: IGFBP3 (1:200, MAB305; R&D Systems, Minneapolis, MN, USA), HIF-1α (1:500, H1alpha67-ChIP Grade; Abcam, Cambridge, UK), HIF-2α (1:1,000, ab8365; Abcam, Cambridge, UK), HO-1 (1:200, GTX101147, Genetex, Irvine, CA, USA), or Von Hippel-Lindau (VHL) (1:200, GTX101087, Genetex, Irvine, CA, USA) at 4 °C overnight (about 17 h).

In ICC, 0.2 × 10^5^ cells were cultured on 25 mm × 25 mm glass slides and incubated under hypoxic or normoxic conditions for 17 h. Then, the slides were incubated with primary antibodies: IGFBP3 (1:200; MAB305; R&D Systems, Minneapolis, MN, USA), HIF-1α (1:300; GTX127309; Genetex, Irvine, CA, USA), or HIF-2α (1:300; GTX30114; Genetex, Irvine, CA, USA) at 4 °C overnight (about 17 h).

A chromogen in IHC and ICC was developed, following the protocol of UltraVision Quanto Detection System HRP DAB (Thermo Fisher Scientific, Waltham, MA, USA). The sections or slides were stained with hematoxylin for examination, and photos were taken using Moticam X3 Plus (Motic, Speed Fair Co., Ltd, HK) microscope camera by Motic Images Plus 3.0 software. The signals of IHC and ICC were quantified and analyzed by ImageJ 1.53 k software (NIH, USA).

### Protein analysis

The total protein from the cells was purified using a Triton X-100 Lysis Buffer (Boston BioProducts, Ashland, MA, USA), and the total protein from the xenograft tumors was purified using a Tissue-PE LB Buffer (GoldBio, St.Louis, MO, USA). These lysis buffers contained Halt Protease and Phosphatase Inhibitor Cocktail (Thermo Fisher Scientific, Waltham, MA, USA). The final protein concentrations were determined using a Bio-Rad Protein Assay Dye Reagent (Bio-Rad, Hercules, CA, USA). Then, the proteins were mixed with a sample buffer T-Pro Laemmli (SDS sample) Reagent (reducing 4 ×) (T-Pro Biotechnology, New Taipei City, Taiwan) in final concentrations of 40 μg per 20 μL and incubated at 100 °C for 10 min. The samples were separated by electrophoresis in a gradient (4 to 15%) SDS-PAGE gel (SMOBIO Technology, Inc. Hsinchu, Taiwan) in a ProSieve EX Running Buffer (Lonza, Basel, Switzerland) with 60 mA for 45 min. Then, they were transferred from the gel to PVDF membranes (0.45 μm. Pall Corporation, New York, NY, USA) by Semi-Dry EBU-4000 Blotting System (Expedeon, Cambridge, UK) in a ProSieve EX Transfer Buffer (Lonza, Basel, Switzerland) with 375 mA for 25 min. The membranes were blocked with Trident Universal Protein Blocking Reagent (Genetex, Irvine, CA, USA) and were incubated with antibodies: IGFBP3 (1:1000; GTX100454; Genetex, Irvine, CA, USA), HIF-1α (1:1000, GTX127309; Genetex, Irvine, CA, USA), HIF-2α (1:1000, GTX30114; Genetex, Irvine, CA, USA), VHL (1:5000, GTX101087, Genetex, Irvine, CA, USA), HO-1 (1:2000, GTX101147, Genetex, Irvine, CA, USA), GAPDH (1:50,000; GTX100118; Genetex, Irvine, CA, USA), cyclophilin A (1:30,000; GTX104698; Genetex, Irvine, CA, USA), or HSP90 (1:100,000; ab32568; Abcam, Cambridge, UK). HSP90 was used as the control. Signals were developed, following the Trident Femto Western HRP Substrate (Genetex, Irvine, CA, USA) manufacturer’s recommendations, and were photographed using UVP BioSpectrum 600 (Analytik Jena AG, Jena, Germany). The signals of Western blots were quantified and analyzed by Image Studio Lite version 5.2 (LI-COR, Lincoln, NE, USA).

### Quantitative real-time PCR and primers

The expressions of IGFBP3, HIF-1α, and HIF-2α mRNA were detected, and GAPDH was used as the normalizing control^27^. The total RNA from P0 and P4 was purified using a Novel Total RNA mini kit (NovelGene Biotech Corporation, Taipei, Taiwan). The reverse transcript to cDNA followed the protocol of SuperScript III Reverse Transcriptase (Thermo Fisher Scientific, Waltham, MA, USA). Gene expressions were analyzed using ABI7900 (Thermo Fisher Scientific, Waltham, MA, USA. Support by Branch Office of Research and Development, NTU MC) with SYBR Green Real-time PCR Master Mix (Toyobo, Osaka, Japan). The specific PCR primer sequences of these genes were as follows: IGFBP3 forward, 5′- TGGGCCATGACTGAGGAAA -3′ and reverse, 5′- TGCCGACCTTCTTGGGTTT -3′; HIF-1α forward, 5′-AGTGCCACATCATCACCATATAGAGAT-3′ and reverse, 5′- CTGTTCTATGACTCCTTTTCCTGCT-3′; HIF-2α forward, 5′- AGCCTCCATCTGCCATCAGTC-3′ and reverse, 5′- CTTGCCATGCCTGACACCTTG-3′; GAPDH forward, 5′- TGGTATCGTGGAAGGACTCA-3′ and reverse, 5′- AGTGGGTGTCGCTGTTGAAG-3′. The quantitative real-time PCR data were analyzed using the 2^−ΔΔCt^ method.

### Promoter regulation study (Luciferase assay)

The promoter fragments of *IGFBP3* (+ 160/ − 1414, + 160, 5′-AAACTCGAGGCATTCGTGTGTACCTCGTG-3′;–1414, 5′-CCCGAGCTCTGATCTTCCCCTGTCCACTC-3′), *HIF-1a* (+ 80/ − 1871, + 80, 5′- AAACTCGAGCTCTCCTCAGGTGGCTTGTC-3′;–1871, 5′- CCCGAGCTCGAGTTGCAGTGAGCCGAAAT-3′), and *HIF-2a* (+ 46/-1222, + 46, 5′-AAACTCGAGGAGGACAAGCTGGCAGAGAC-3′;–1222, 5′- CCCGAGCTCGTGTTCCGCATTTTGGAAGT-3′) were generated by chromosomal DNA extraction from P0, amplified by Q5 High-Fidelity DNA Polymerase ((NEB, Ipswich, MA, USA), and constructed into pGL3 (Promega, Madison, WI, USA). The pGL3-IGFBP3, pGL3-HIF-1α, pGL3-HIF-2α, or empty pGL3 plasmids were transiently transfected into P0 and P4 (500 ng plasmid per 5 × 10^4^ cells) and incubated for 16 h. Recombinant IGFBP3 proteins (50 ng/mL; 675-B3; R&D Systems, Minneapolis, MN, USA) or IGFBP3 neutralizing antibodies (5 ng/mL; MAB305; R&D Systems, Minneapolis, MN, USA) were added and cultured in hypoxic or normoxic conditions for 16 h. FLUOstar Omega (BMG LABTECH, Offenburg, Germany) measured the luciferase activities, following the protocol of Luc-Pair Firefly Luciferase HS Assay Kit (Genecopoeia, Rockville, MD, USA). Luciferase activities were calculated relative to the empty pGL3 transfectants.

### Human angiogenesis antibody array

The expression of angiogenesis-related proteins among P0, P4, P4-I, and P4-V under normoxic and hypoxic conditions (17 h) were identified using Human Angiogenesis Antibody Array-Membrane (43 targets) (Abcam, Cambridge, UK). The membrane blots were photographed using iBright CL1000 Western Blot Imaging Systems (Thermo Fisher Scientific, Waltham, MA, USA) and analyzed by Image Studio Lite version 5.2 (LI-COR, Lincoln, NE, USA).

### Approval

The animal study protocol had been reviewed and approved by the Institutional Animal Care and Use Committee(IACUC) , National Taiwan University College of Medicine and College of Public Health (Approval Number: 20130524).

The animal study was carried out in accordance with IACUC guidelines and regulations, and compliance with the ARRIVE guidelines.

The research plan containing the gene recombination and transfection of the plasmids was approved by National Taiwan University Hospital and was carried out according to National Taiwan University Hospital's gene recombination and biological materials experimental regulations.

### Ethics approval and consent to participate

IACUC Approval No: 20130524. The animal use protocols listed below had been reviewed and approved by the Institutional Animal Care and Use Committee(IACUC), National Taiwan University College of Medicine and College of Public Health.

## Supplementary Information

Supplementary Information.

## Data Availability

All data generated and analysed during this study are included in the article. Cell lines and reagents are commercially available or available from the corresponding author on reasonable request.
