# Supplementary material for: Differential expression of hypoxia-inducible factors related to the invasiveness of epithelial ovarian cancer
Source: Sci Rep. 2021 Nov 25;11:22925. doi: 10.1038/s41598-021-02400-1 (PMC8616920; doi:10.1038/s41598-021-02400-1)

Differential Expression of Hypoxia-inducible factors Related to the Invasiveness of Epithelial Ovarian Cancer

Ho-Jun Shih, Hsin-Fang Chang, Chi-Ling Chen, Pao-Ling Torng

Supplementary Figure 1. Western blots. (a)Western blot of GAPDH, Cyclophilin A, HSP90, and α-tubulin in gradient (4 to 15%) SDS-PAGE gel. Thermo Scientific pageruler Cat. No. 26616 protein ladder (10~180 kDa) was used as standard. (b) The non-specific binding signal of HIF-2α (between 15 and 70 kDa) may interfere with signals of examined proteins, such as IGFBP3 (42 to 44 kDa), HO-1 (35 kDa), and VHL (24 kDa). Therefore, to avoid interfering with non-specific binding signals and protein loss during the stripping process, membranes after transferred were cropped separately into ranges of molecular weights. The cropped membranes were then incubated with corresponding antibodies. Membrane with molecular weight size above 70 kDa was incubated with HIF-1α or HIF-2α. Membrane with molecular weight between 40 and 70 kDa was incubated with IGFBP3. And membrane with molecular weight under 40 kDa was incubated with HO-1or VHL. All membranes were stripped ones and incubated with internal control: HSP90 (above 70kDa), GAPDH, or Cyclophilin A. During the cell subculture process, the excess cells were collected and mixed, and the proteins were extracted for operational control, labeled as Mix of all the cells. Photoshop CS2 version 9 was used to assemble the figure.

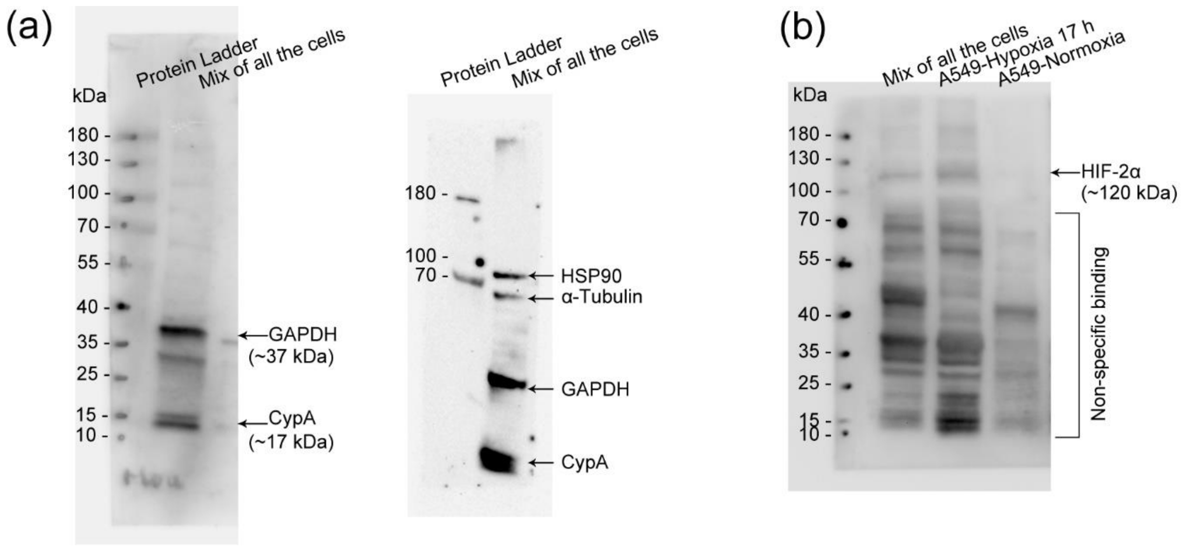

Supplementary Figure 2. Protein expression analysis of xenograft tumors. Raw data representing the corresponding Western blot results of xenograft tumors as shown in Figure 2. (a) IGFBP3 expressions in xenograft tumors. (b) HIF-1α and HIF-2α expression in xenograft tumors. CypA as internal control. Photoshop CS2 version 9 was used to assemble the figure.

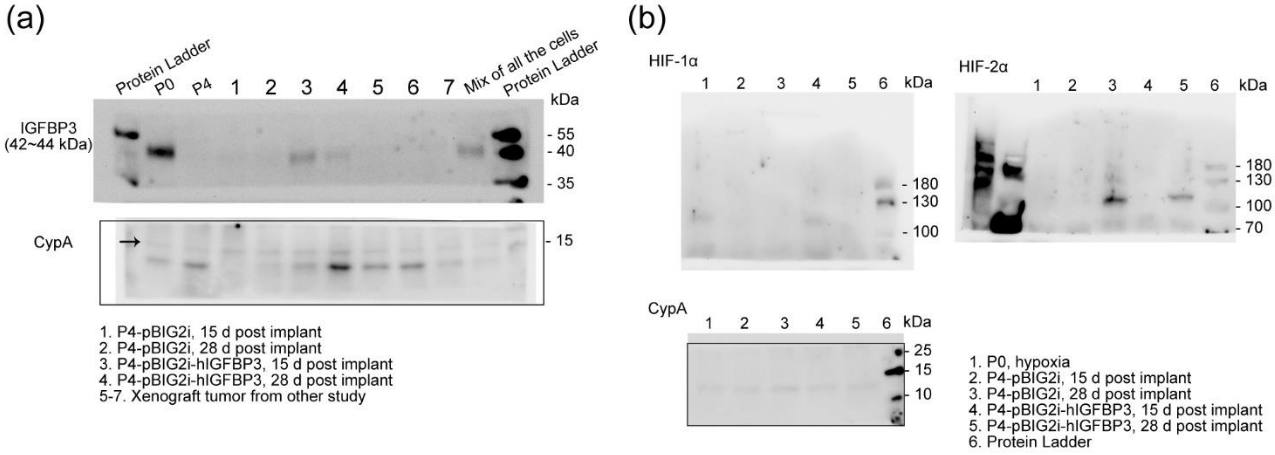

Supplementary Figure 3. Protein expression analysis of cell cultures under normoxic and hypoxic conditions. (A)Raw data of the western blot results as shown in Figure 4. Membranes after transferred were cropped before 1st antibody hybridization. The arrow (→) indicates the location of the protein, the line segment ([ ) indicates the edge of the membranes. Photoshop CS2 version 9 was used to assemble the figure. The molecular weight label by LuminolPen.

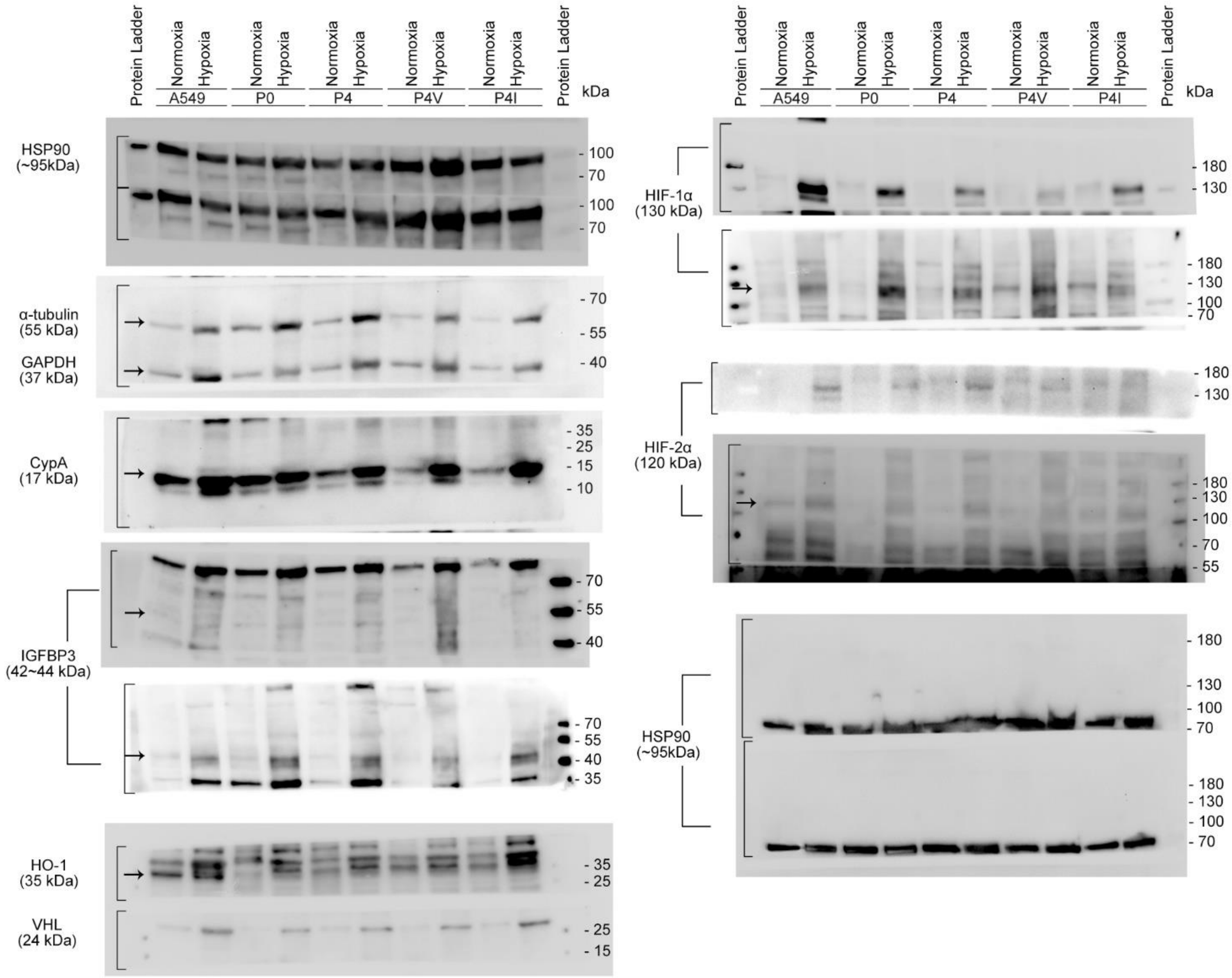

Supplementary Figure 4. Tumor growth in SCID mice breeding P4-pBIG2i and P4-pBIG2i-hIGFBP3 transfectants. Tumors were treated with 2 mg/mL of doxycycline at day 9 (▼) when tumor size reached to 0.5 cm in diameter. P4-pBIG2i implant grew fast without doxycycline stimulation, but it showed growth arrest after doxycycline feeding (P4-pBIG2i + doxycycline). There were no differences in tumor growth between P4-pBIG2i treated with doxycycline and P4-pBIG2i-hIGFBP3 transplants (both tumors with low IGFBP3 expression). Tumor growth was slowest in P4-pBIG2i-IGFPB3 implant upon IGFBP3 stimulation (tumor with high IGFBP3 expression). Excel 2016 were used to generate figure.

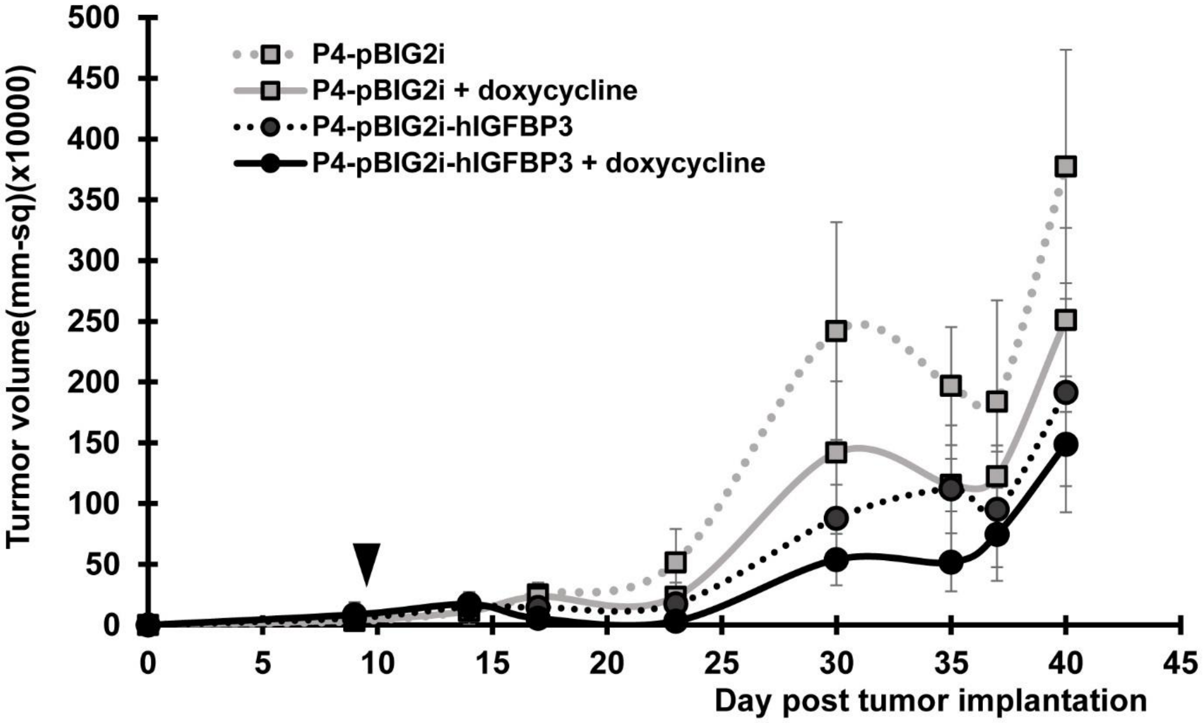

Supplement: Supplementary file 1 — Supplementary Information. [file 41598_2021_2400_MOESM1_ESM.pdf]
